# Comparison of the clinical impact of 2-[^18^F]FDG-PET and cerebrospinal fluid biomarkers in patients suspected of Alzheimer’s disease

**DOI:** 10.1371/journal.pone.0248413

**Published:** 2021-03-12

**Authors:** Le Gjerum, Birgitte Bo Andersen, Marie Bruun, Anja Hviid Simonsen, Otto Mølby Henriksen, Ian Law, Steen Gregers Hasselbalch, Kristian Steen Frederiksen

**Affiliations:** 1 Department of Neurology, Danish Dementia Research Centre, Rigshospitalet, University of Copenhagen, Copenhagen, Denmark; 2 Department of Clinical Physiology, Nuclear Medicine & PET, Rigshospitalet, University of Copenhagen, Copenhagen, Denmark; Fondazione Istituto G.Giglio di Cefalu, ITALY

## Abstract

**Background:**

The two biomarkers 2-[^18^F]FDG-PET and cerebrospinal fluid biomarkers are both recommended to support the diagnosis of Alzheimer’s disease. However, there is a lack of knowledge for the comparison of the two biomarkers in a routine clinical setting.

**Objective:**

The aim was to compare the clinical impact of 2-[^18^F]FDG-PET and cerebrospinal fluid biomarkers on diagnosis, prognosis, and patient management in patients suspected of Alzheimer’s disease.

**Methods:**

Eighty-one patients clinically suspected of Alzheimer’s disease were retrospectively included from the Copenhagen Memory Clinic. As part of the clinical work-up all patients had a standard diagnostic program examination including MRI and ancillary investigations with 2-[^18^F]FDG-PET and cerebrospinal fluid biomarkers. An incremental study design was used to evaluate the clinical impact of the biomarkers. First, the diagnostic evaluation was based on the standard diagnostic program, then the diagnostic evaluation was revised after addition of either cerebrospinal fluid biomarkers or 2-[^18^F]FDG-PET. At each diagnostic evaluation, two blinded dementia specialists made a consensus decision on diagnosis, prediction of disease course, and change in patient management. Confidence in the decision was measured on a visual analogue scale (0–100). After 6 months, the diagnostic evaluation was performed with addition of the other biomarker. A clinical follow-up after 12 months was used as reference for diagnosis and disease course.

**Results:**

The two biomarkers had a similar clinical value across all diagnosis when added individually to the standard diagnostic program. However, for the correctly diagnosed patient with Alzheimer’s disease cerebrospinal fluid biomarkers had a significantly higher impact on diagnostic confidence (mean scores±SD: 88±11 vs. 82±11, p = 0.046) and a significant reduction in the need for ancillary investigations (23 vs. 18 patients, p = 0.049) compared to 2-[^18^F]FDG-PET.

**Conclusion:**

The two biomarkers had similar clinical impact on diagnosis, but cerebrospinal fluid biomarkers had a more significant value in corroborating the diagnosis of Alzheimer’s disease compared to 2-[^18^F]FDG-PET.

## Introduction

Diagnosing Alzheimer’s disease (AD) can be challenging due to clinical heterogeneity as well as a considerable overlap between various subtypes of dementia [1–3]. The clinical practice for diagnosing dementia has gradually shifted from a solely clinical-based diagnosis to a biomarker-supported diagnosis as the understanding of disease-specific biomarkers for AD have advanced greatly and the biomarkers have become available in clinical settings. The growing body of evidence for the utility of biomarkers across the spectrum of AD has led to proposal of the A/T/N classification scheme as a biomarker-based research framework for staging of AD pathophysiology [4]. In the framework, the AD biomarkers were divided into three binary categories based on the specific underlying pathophysiology that the biomarkers measure: “A” refers to measure of brain Aβ deposition with cerebrospinal fluid (CSF) Aβ1–42 (Aβ42) and amyloid positron emission tomography (PET); “T” refers to measure of tau pathology with CSF phosphorylated tau at threonine 181 (p-tau); and “N” refers to measures of AD-like neurodegeneration with CSF total tau (t-tau), atrophy on structural magnetic resonance imaging (MRI), and hypometabolism on PET using 2-[^18^F]fluoro-2-deoxy-D-glucose (2-[^18^F]FDG) as tracer [5]. CSF biomarkers are easy accessible biomarkers to support a diagnosis of AD in many clinical centers [6], nevertheless 2-[^18^F]FDG-PET has also reached a prominent role in the early differential diagnosis of AD in both clinical and research settings [7, 8]. Previous studies have shown that 2-[^18^F]FDG-PET can be valuable to distinguish neurodegenerative dementias from other forms of cognitive dysfunction, and detection of distinctive disease patterns of hypometabolism can support the diagnosis of AD, frontotemporal dementia (FTD), and dementia with Lewy bodies (DLB) [9–13].

Biomarkers are recommended as addition to the clinical diagnostic evaluation, if the diagnosis is uncertain or the clinical presentation is atypical to improve the diagnostic accuracy [10, 14]. However, the clinical value of biomarkers is outlined in general terms and there is a lack of recommendations for prioritizing and weighing of the biomarkers in the differential diagnosis of dementia. This emphasizes the need for further research to optimize and validate the clinical impact of biomarkers in decision making of dementia.

Notwithstanding the emerging role of 2-[^18^F]FDG-PET and CSF biomarkers in clinical diagnosis of AD, a head-to-head comparison of the diagnostic utility of 2-[^18^F]FDG-PET and CSF biomarkers have predominantly been investigated in mild cognitive impairment (MCI) populations focusing on conversion to AD dementia [15–23], whereas the knowledge of added value in the differential diagnosis of AD in a clinical setting are limited [24, 25]. Moreover, a recent systematic review of the biomarker accuracy for AD concluded that both 2-[^18^F]FDG-PET and CSF biomarkers may add accuracy to clinical evaluation of older adults with dementia, however, also concluded that there is sparse knowledge of the impact of 2-[^18^F]FDG-PET and CSF biomarkers in clinical decision making such as additional testing [26].

The objective was to evaluate the clinical impact of 2-[^18^F]FDG-PET and CSF biomarkers on diagnosis, prognosis, and patient management in the differential diagnosis of AD in patients where the diagnostic certainty was low following the standard diagnostic program.

## Materials and methods

### Study population

We retrospectively selected 81 patients from an initial sample of 210 patients included between March 2015 and June 2016 from the Copenhagen Memory Clinic at Rigshospitalet in Denmark as part of the PredictND study (S1 Fig). The criteria for retrospective inclusion were: 1) Patients suspected of possible AD at baseline with clinical indication of addition of 2-[^18^F]FDG-PET and CSF biomarkers to the standard diagnostic program deemed by the clinician to increase certainty of the diagnosis; 2) MRI, 2-[^18^F]FDG-PET, and CSF biomarkers performed as part of their diagnostic work-up for dementia. The exclusion criteria were any clinical knowledge of the patient by the clinicians in the consensus group.

The patients in the PredictND study were prospectively included if they were suspected of having cognitive complaints due to neurodegenerative disease, and had a baseline mini-mental state examination (MMSE) score ≥ 18, a clinical dementia rating (CDR) global score ≤ 1.0, and had undergone a T1-weighted MRI ≥ 1.5 Tesla. They were excluded if they had excessive alcohol intake and/or substance abuse within the last 2 years or had a psychiatric or brain disorder diagnosis that could have cause the cognitive impairment. The patients had a standardized clinical follow-up after 12 months as part of the PredictND study [27].

### Clinical examinations and supplementary investigations

In accordance with the current recommendations for diagnosis and assessment of dementia in a clinical setting, each patient was assessed with a standard diagnostic program including medical history, physical and neurological examinations, neuropsychological assessment, routine blood screening, and MRI as part of the routine clinical evaluation [7, 14, 28]. The neuropsychological assessment included MMSE, Addenbrooke’s cognitive examination, CERAD (consortium to establish a registry for Alzheimer’s disease) word list and delayed recall, digit span forward and backward, trail making test A and B, and animal fluency test.

In this study, all patients underwent 2-[^18^F]FDG-PET and CSF biomarkers due to suspected possible AD and the clinician found indication for additional biomarkers to the standard diagnostic program to increase the diagnostic accuracy.

Additional investigations of dopamine transporter single photon emission computed tomography (DAT-SPECT) (n = 8) and psychiatric assessment (n = 4) were performed on clinical indication. A subgroup of the patients underwent amyloid PET imaging (n = 8), but these test results were not included in this study.

The study was approved by the Danish National Committee on Health Research (no. H-1-2014-126). All patients provided written informed consent for their data to be used for research purposes.

### Study design

We used an incremental study design to evaluate the clinical impact of 2-[^18^F]FDG-PET and CSF biomarkers. The evaluation was done by two clinicians (either BBA and KSF or SGH and KSF), who were dementia specialists with several years of experience with the diagnostic use of CSF biomarkers and 2-[^18^F]FDG-PET. The patients’ baseline data were retrospectively and blindly assessed by the two clinicians. The imaging results were presented as clinical written reports but could be visually assessed on request to mimic the clinical diagnostic work-up.

**Fig 1:**
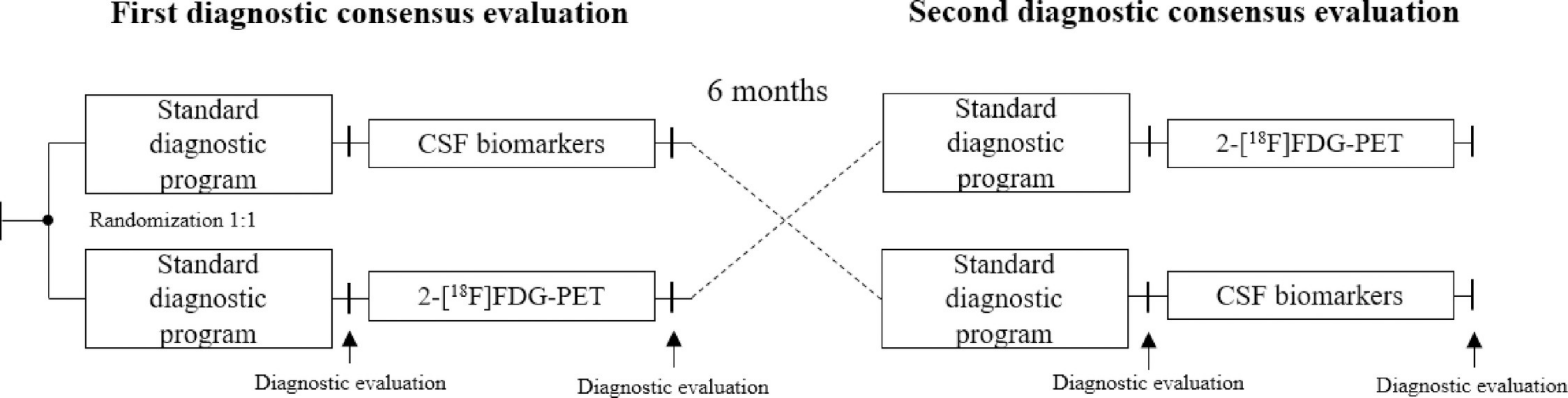
Flow diagram of the three-step incremental evaluation. Abbreviations: AD: Alzheimer’s disease; CSF: cerebrospinal fluid; standard diagnostic program: medical history, physical and neurological examinations, cognitive testing, routine blood screening, and MRI; 2-[^18^F]FDG-PET: 2-[^18^F]Fluoro-2-deoxy-D-glucose positron emission tomography;The stepwise diagnostic consensus evaluation was done by two blinded dementia specialists. In the first step, the evaluation was based solely on the standard diagnostic program. In the second step, the evaluation was based on the additional biomarker (CSF biomarkers or 2-[^18^F]FDG-PET). After 6 months, diagnostic consensus evaluation was done on the same patients in the stepwise process with addition of the other biomarker in the second step.

In the first step (standard diagnostic program), the diagnostic evaluation was based on the patients’ baseline data except CSF biomarkers and 2-[^18^F]FDG-PET. In the second step (standard diagnostic program + biomarker), the diagnostic evaluation was revised based on addition of either CSF biomarkers or 2-[^18^F]FDG-PET. After 6 months, the same two clinicians evaluated the same patients with addition of the other biomarker in the second step. The order of the added biomarker (either CSF biomarkers or 2-[^18^F]FDG-PET) was randomized (Fig 1).

At each diagnostic evaluation, the clinicians reached a consensus decision on (1) diagnosis and confidence in the diagnosis reported as a visual rating scale (VAS) score between 0–100; (2) prediction of disease course after 12 months reported as stable or progressed, and confidence in the prediction of disease course was reported as a VAS score between 0–100; (3) change in anti-dementia medication, for instance cholinesterase inhibitors; (4) change in psychosocial interventions including assignment of a case manager, speech therapy, or physiotherapy; and (5) need for ancillary investigations, for instance CSF biomarkers, 2-[^18^F]FDG-PET, or other supplementary investigations such as DAT-SPECT, PET amyloid scan, electroencephalography, or psychiatric assessment.

The reference diagnosis and disease course were determined by an experienced dementia specialist based on the clinical impression at a 12 months follow-up visit including clinical interview with the patient, assessment of MMSE and CDR, and all available clinical patient data as described in [27].

### Diagnostic criteria

At baseline, patients were diagnosed with a syndrome diagnosis of either MCI (n = 19) or dementia (n = 48) based on the National Institute of Aging-Alzheimer’s Association criteria [10, 29]. Patients were diagnosed with subjective cognitive decline (SCD) (n = 14), if the cognitive complaints were present without confirmed objective cognitive impairment, and the criteria for MCI and dementia were not met [30]. Patients with dementia or MCI were diagnosed with an underlying etiology diagnosis according to the following diagnostic criteria: the NIA-AA guidelines for AD [10] (n = 31), McKhann criteria for FTD [31] (n = 2) with Rascovsky criteria for behavioural variant FTD and Gorno-Tempini criteria for semantic dementia and progressive non-fluent aphasia [11, 12], McKeith criteria for DLB [13] (n = 3), VASCOG criteria for VaD [32] (n = 4), and the IWG-2 criteria for mixed AD [9] (n = 1). The etiology was classified as “other diagnosis” for other causes of cognitive disorders than those stated above (e.g. Parkinson’s disease with dementia, atypical Parkinsonism, or normal pressure hydrocephalus) (n = 26).

### Magnetic resonance imaging

MRI was performed on a clinical 1.5 or 3.0 Tesla system. The scan included a three-dimensional T1-weighted magnetization-prepared rapid acquisition gradient-echo (T1-MPRAGE) sequence and a T2-weighted fluid attenuation inversion recovery sequence. The T1-MPRAGE sequence was segmented using FreeSurfer (version 5.3.0) [33] to generate a hippocampal volumetric report which contained the absolute hippocampal volume, hippocampal volume relative to intracranial volume and compared this to a database of healthy elderly subjects giving a relative comparison of the individual patient and a comparison population [34]. Each scan was evaluated by an experienced neuroradiologist in a clinical written MRI report as part of the clinical routine. The hippocampal volumetric report and the clinical written MRI report were used by the clinician as part of the diagnostic evaluation.

### Cerebrospinal fluid

The CSF biomarkers (Aβ42, total tau, and p-tau) were analyzed with enzyme-linked immunosorbent assay using commercially available kits (Innotest, Fujirebio, Europe, Ghent, Belgium). CSF biomarkers were considered as abnormal based on the local clinical cut off-values for each biomarker, i.e. Aβ42 values <550 pg/mL, p-tau levels >80 pg/mL, and t-tau >400 pg/mL [35].

### 2-[^18^F]fluoro-2-deoxy-D-glucose positron emission tomography

The PET images were performed according to the international practice guideline [36]. The PET images were acquired using clinical PET/CT or PET/MRI systems after an intravenous bolus of 200–300 MBq 2-[^18^F]FDG as a 10 min static scan during the 40–60 min post-injection time window. The 2-[^18^F]FDG-PET image was co-registered to the structural scan (i.e. low-dose CT, non-enhanced diagnostic CT or MRI) in a clinical standardized reading approach in SyngoVia (SyngoVia, MI Neurology, Siemens Healthcare, Erlangen, Germany). The PET image was analyzed using clinically routine quantification methods, i.e. the statistical parametric mapping and statistical surface projects were performed using Scenium in comparison with a database of healthy subjects together with a visual inspection. The 2-[^18^F]FDG-PET image was evaluated by an experienced nuclear medicine physician in a written report as part of the clinical routine.

### Data analysis

Differences in baseline values between the two groups were assessed using unpaired t-test for continuous values and Fisher’s exact test for categorical values. The inter-rater concordance on the etiological diagnosis (AD group or non-AD group) of the two baseline evaluations without biomarkers was assessed using the unweighted Cohen’s κ coefficient with 95% confidence intervals (CI) [37]. The accuracies of diagnosis and prediction of disease course were evaluated by comparing the baseline consensus evaluation to the reference evaluation at 12 months follow-up.

McNemar’s test was used to evaluate the paired comparison between the baseline diagnoses, prediction of disease course, change in anti-dementia medication and psychosocial interventions, and need for further investigations. Unpaired t-tests were used to evaluate the impact of biomarkers on continuous variables (confidence for diagnosis and disease course).

Statistical analysis was performed using SAS Studio software, version 9.4 (SAS Institute Inc., Cary, NC, USA). A two-sided p-value<0.05 was considered indicative of statistical significance.

## Results

### Study population

At 12 months clinical follow-up, 1 (7%) patient diagnosed with SCD at baseline had progressed to MCI due to AD, and seven (37%) patients with a syndrome diagnosis of MCI at baseline had progressed to AD dementia (n = 5) and non-AD dementia (n = 2), whereas 12 (63%) patients had a diagnosis of MCI.

Based on the etiological diagnosis at 12 months clinical follow-up, the 81 patients were divided into an AD group (n = 37) consisting of patients diagnosed with probable AD (n = 34), atypical AD (n = 1), and mixed AD (n = 2); and non-AD (n = 44) consisting of patients diagnosed with DLB (n = 5), FTD (n = 3), VaD (n = 6), other diagnosis (n = 15), and SCD (n = 15).

At baseline, we found that the AD group had lower mean MMSE scores, lower median Aβ42 values and higher median tau values than the non-AD group (Table 1). Three of the patients (1 in the AD group and 2 in the non-AD group) were not examined with additional neuropsychological tests. We have included an informative table on the clinical data of the included patients from the retrospective cohort and patients from the PredictND cohort (S1 Table).

**Table 1:** Baseline demographics.

	AD group	Non-AD group
**Patients, n**	37	44
**Female, n (%)**	22 (59)	18 (41)
**Age, mean years ±SD**	70.3±9.3	68.1±9.2
**Education, mean years ±SD**	13.6±2.9	12.8±2.7
**Symptom duration, mean years ±SD**	2.3±1.9	2.8±2.9
**CDR global score, n (0/0.5/1.0)**	7/17/11^a^	7/23/11^b^
**MMSE, mean score ±SD**	26.1±2.8	27.5±2.7*
**ACE, mean score ±SD**	77.9±10.2	83.3±10.6*
**CSF Aβ42, median volume pg/mL [IQR]**	528 [148]	851 [420]*
**CSF total tau, median volume pg/mL [IQR]**	556 [357]	240 [247]*
**CSF p-tau, median volume pg/mL [IQR]**	72 [33]	41 [31]*

Abbreviations: Aβ42: amyloid beta 1–42; ACE: Addenbrooke’s cognitive examination; AD: Alzheimer’s disease; CDR: clinical dementia rating; CSF: cerebrospinal fluid; IQR: interquartile range; MMSE: mini-mental state examination; n: number; p-tau: phosphorylated tau at threonine 181; SD: standard deviation.

* Differ significantly from AD group (p <0.05).

^a^ Data missing for 2 patients in the AD group.

^b^ Data missing for 3 patients in the non-AD group.

### Inter-rater agreement

The inter-rater concordance on the diagnosis showed a substantial agreement at the two diagnostic evaluation based on the standard diagnostic program, i.e. without biomarkers (90%, κ = 0.80, 95% CI 0.67–0.93).

### Clinical impact of 2-[^18^F]FDG-PET and CSF biomarkers on diagnosis

The diagnose at 12 months clinical follow-up was used as reference diagnosis. As shown in Table 2, addition of 2-[^18^F]FDG-PET to the standard diagnostic program led to a change in diagnosis for 4 patients (5%) (1 correct and 3 incorrect), and overall 65 patients (80%) were correctly classified with a high accuracy in the AD group (89%) and moderate accuracy in the non-AD group (77%). In comparison, addition of CSF biomarkers to the standard diagnostic program led to a change in diagnosis for 12 patients (15%) (7 correct and 5 incorrect), and overall 72 patients (89%) were correctly classified with a high accuracy in the AD group (95%) and non-AD group (84%). The diagnostic confidence for the correctly classified patients in the AD group was significantly higher after addition of 2-[^18^F]FDG-PET (74±8 vs. 82±11, p = 0.0006) and CSF biomarkers (73±11 vs. 88±11, p < 0.0001) to the standard diagnostic program. A direct comparison between the two biomarkers showed that addition of CSF biomarkers led to a significantly higher diagnostic confidence for correctly classified patients as compared to addition of 2-[^18^F]FDG-PET (p = 0.046).

**Table 2:** The clinical impact of 2-[^18^F]FDG-PET and CSF biomarkers on diagnosis and confidence in diagnosis.

According to 12 months follow-up diagnosis, n (%)	AD group		Non-AD group	
	Correct diagnosis	Incorrect diagnosis	Correct diagnosis	Incorrect diagnosis
Standard diagnostic program + 2-[^18^F]FDG-PET	33 (89)	4 (11)	32 (77)	12 (27)
Change in diagnosis from standard diagnostic program	0	1	1	2
Standard diagnostic program + CSF biomarkers	35 (95)	2 (5)	37 (84)	7 (16)
Change in diagnosis from standard diagnostic program	2	2	5	3
Diagnostic confidence, mean VAS score ±SD				
Standard diagnostic program + 2-[^18^F]FDG-PET	82±11	63±18	76±15	66±10
Change in diagnostic confidence from standard diagnostic program	9±7^**×**^	-10±22	5±8	1±7
Standard diagnostic program + CSF biomarkers	88±11*	68±25	73±14	74±16
Change in diagnostic confidence from standard diagnostic program	20±13^×^*	3±4	4±14	12±21

Abbreviations: AD: Alzheimer’s disease; CSF: cerebrospinal fluid; n: number; SD: standard deviation; standard diagnostic program: medical history, physical and neurological examinations, cognitive testing, routine blood screening, and MRI; VAS: visual rating scale; 2-[^18^F]FDG-PET: 2-[^18^F]Fluoro-2-deoxy-D-glucose positron emission tomography

^**×**^Statically significant difference as compared to standard diagnostic program (p <0.05).

*Statically significant difference as compared to 2-[^18^F]FDG-PET (p <0.05).

A subgroup analysis including patients diagnosed with AD (n = 37), DLB (n = 5), and FTD (n = 3) showed that addition of CSF biomarkers to the standard diagnostic program led to a higher accuracy with a significantly higher diagnostic confidence when compared to addition of 2-[^18^F]FDG-PET (S2 Table). Addition of 2-[^18^F]FDG-PET and CSF biomarkers to the standard diagnostic program did not change the accuracy for DLB and FTD.

### Clinical value of 2-[^18^F]FDG-PET and CSF biomarkers on predicting disease course

The disease course determined by an experienced dementia specialist based on the clinical impression at 12 months clinical follow-up were used as reference. At 12 months follow-up, 37 of the patients with either MCI or dementia remained stable and 29 patients had progressed. The total number of correctly classified patients with regards to disease course was comparable after addition of CSF biomarkers and 2-[^18^F]FDG-PET. Addition of the biomarkers did not improve the clinician’s ability to predict the disease course nor the confidence in the predicted disease course (Table 3).

**Table 3:** The clinical impact of 2-[^18^F]FDG-PET and CSF biomarkers on disease course and confidence in disease course for patients with MCI and dementia.

According to disease course at 12 months follow-up, n (%)	Correct	Incorrect
Standard diagnostic program + 2-[^18^F]FDG-PET	37 (56)	29 (44)
Change in disease course from standard diagnostic program	2	1
Standard diagnostic program + CSF biomarkers	34 (52)	32 (48)
Change in disease course from standard diagnostic program	4	5
Diagnostic confidence, mean VAS score ±SD		
Standard diagnostic program + 2-[^18^F]FDG-PET	71±13	68±13
Change in confidence from standard diagnostic program	1±13	5±13
Standard diagnostic program + CSF biomarkers	69±12	72±10
Change in confidence from standard diagnostic program	3±12	6±11^**×**^

Abbreviations: AD: Alzheimer’s disease; CSF: cerebrospinal fluid; MCI: mild cognitive impairment; n: number; SD: standard deviation; standard diagnostic program: medical history, physical and neurological examinations, cognitive testing, routine blood screening, and MRI; VAS: visual rating scale; 2-[^18^F]FDG-PET: 2-[^18^F]Fluoro-2-deoxy-D-glucose positron emission tomography

^**×**^Statically significant difference as compared to standard diagnostic program (p <0.05).

### Impact of CSF biomarkers and 2-[^18^F]FDG-PET on patient management

As shown in Table 4, addition of 2-[^18^F]FDG-PET and CSF biomarkers were associated with a significant change in anti-dementia medication and psychosocial interventions when compared to the standard diagnostic program, and the change was similar between the two biomarkers.

**Table 4:** Change in patient management following 2-[^18^F]FDG-PET and CSF.

Need for anti-dementia medication, n (%)	
Number of patients where anti-dementia medication was prescribed following 2-[^18^F]FDG-PET	12 (15)^**×**^
Number of patients where anti-dementia medication was prescribed following CSF biomarkers	16 (20)^**×**^
Need for care, n (%)	
Number of patients where care was instituted following 2-[^18^F]FDG-PET	14 (17)^**×**^
Number of patients where care was instituted following CSF biomarkers	15 (19)^**×**^
Need for ancillary investigations, n (%)	
Number of patients in which ancillary investigations were necessary following 2-[^18^F]FDG-PET	18 (22)^**×**^
Number of patients in which ancillary investigations were necessary following CSF biomarkers	23 (28)^**×**^

Abbreviations: AD: Alzheimer’s disease; CSF: cerebrospinal fluid; n: number; standard diagnostic program: medical history, physical and neurological examinations, cognitive testing, routine blood screening, and MRI; 2-[^18^F]FDG-PET: 2-[^18^F]Fluoro-2-deoxy-D-glucose positron emission tomography

^**×**^Statically significant difference as compared to standard diagnostic program (p <0.05).

*Statically significant difference as compared to 2-[^18^F]FDG-PET (p <0.05).

Addition of 2-[^18^F]FDG-PET and CSF biomarkers led to a significantly reduction in the need for ancillary investigations when compared to the standard diagnostic program, and the change was significantly higher for CSF biomarkers as compared to 2-[^18^F]FDG-PET (23 vs. 18 patients, p = 0.049)

## Discussion

In this study, we evaluated and compared the clinical impact of two commonly used supportive diagnostic biomarkers, CSF biomarkers and 2-[^18^F]FDG-PET, in an incremental study design to mimic the clinical practice. The evaluation was performed in patients with 12 months clinical follow-up and clinical indication for addition of 2-[^18^F]FDG-PET and CSF biomarkers to the standard diagnostic program as a result of uncertainty of the clinician in the AD diagnosis. The two biomarkers had similar impact on clinical diagnosis, but CSF biomarkers had a higher impact on diagnostic confidence and led to a reduced need for ancillary investigations compared to 2-[^18^F]FDG-PET.

A few studies have directly compared the diagnostic value of 2-[^18^F]FDG-PET and CSF biomarkers in the differential diagnosis of AD, and overall found a high accuracy for the two biomarkers in the differential diagnosis of AD [16, 18, 19, 24, 25]. These studies showed some discrepancy with regard to the impact of the biomarkers. Two studies found higher accuracies for 2-[^18^F]FDG-PET in diagnosing AD compared to CSF biomarkers [16, 25], whereas two studies concluded the opposite [18, 19], and one study found that the accuracy of the biomarkers depended on the disease stage [24]. The discrepancies may be explained by a number of different factors, such as differences in the included population, variance in operating procedures and biomarker measurement methods and interpretation [38]. In contrast to the previous studies, our study also evaluated the clinical impact after addition of a biomarker to the standard diagnostic program to mimic the use of biomarkers in clinical routine.

In this study, we compared the clinical impact of 2-[^18^F]FDG-PET and CSF biomarkers as applied in a routine clinical setting, i.e. the PET images were analyzed using clinical routine quantification methods together with visual inspection and added to the diagnostic program. The change in diagnosis was higher for addition of CSF biomarkers to the standard diagnostic program as compared to addition of 2-[^18^F]FDG-PET (12 patients vs. 4 patients), and addition of CSF biomarkers to the standard diagnostic program had a higher impact in the non-AD group with change in diagnosis for 8 patients (18%) (5 correct and 3 incorrect), which may indicate that CSF biomarkers have a substantial clinical impact in rule-out AD. Interestingly, the addition of 2-[^18^F]FDG-PET had limited clinical impact on non-AD diagnoses such as DLB and FTD (S2 Table), although specific metabolic patterns have been shown to distinguish between AD, DLB, and FTD [8]. In comparison, a previous study found a specificity greater than 95% in the differential diagnosis of AD towards other dementias based on 2-[^18^F]FDG-PET diagnosis in a study cohort of early-onset AD and other dementias (mean age of 60±4 years) [39].

The lower diagnostic accuracy for 2-[^18^F]FDG-PET in our study may be caused by an overlap of the AD-like patterns of temporoparietal hypometabolism between AD and cerebrovascular disease [5] and the lack of distinct, hypometabolic patterns for non-AD including VaD and NPH.

Overall, the diagnostic confidence increased significantly for correctly classified patients in the AD group after addition of one of the biomarkers, which indicate that the predominant clinical impact after addition of either 2-[^18^F]FDG-PET or CSF biomarkers was to confirm this diagnosis.

This is in line with the recommendations for 2-[^18^F]FDG-PET [8, 40, 41] and corroborated by previous studies demonstrating that the clinical impact of 2-[^18^F]FDG-PET was highest when prior diagnostic confidence was low [42–44].

Addition of either 2-[^18^F]FDG-PET or CSF biomarkers to the standard diagnostic program did not improve the clinician’s ability to predict the disease course (Table 3). This finding was somewhat unexpected, since previous studies have identified different regional patterns of hypometabolism on 2-[^18^F]FDG-PET [21, 45–51] and abnormal level of CSF biomarkers [52–56] as predictors of cognitive decline in dementia and conversion from MCI to dementia. Several studies have compared 2-[^18^F]FDG-PET and CSF biomarkers head-to-head as predictors of cognitive decline and conversion to dementia in MCI patients. Overall, the studies found that CSF biomarkers were highly sensitive for cognitive decline [57], whereas 2-[^18^F]FDG-PET [15, 21, 22, 57] or combinations of 2-[^18^F]FDG-PET and CSF biomarkers [17, 20, 23, 58] were good predictors of conversion to AD dementia based on biomarker positivity. However, these findings may not be very applicable to our study as most of our cohort consisted of patients with dementia.

The low accuracy of both biomarkers to predict disease course may be caused by various factors. The most important factors could be that a follow-up of 12 months may be too short to encompass the complexity of progression, and that our patient population may be too small and heterogenous to pick up predictive power on a group level [59]. Furthermore, CSF Aβ42 and p-tau are markers for staging of AD pathophysiology, and less useful to stage the disease progression in non-AD [5]. Finally, the definitions of how to evaluate cognitive decline is variable and difficult to compare across studies [6].

Our third finding was that the impact of 2-[^18^F]FDG-PET and CSF biomarkers on patient management was similar regarding changes in anti-dementia medication and psychosocial interventions. In line with previous studies, the clinicians were more likely to suggest initiation of anti-dementia medication after addition of a biomarker [42, 43, 60]. A previous study reported that addition of CSF biomarkers had an impact on patient management for 13% of patients including inclusion in clinical trials, intensive follow-up and imaging studies [61], but apart from that the knowledge of the impact of CSF biomarkers on patients management is very limited.

A few studies have evaluated the impact of 2-[^18^F]FDG-PET on patient management and found that changes in anti-dementia medication varied from 17% to 32% of the patients, and the request for ancillary investigations were reduced for 42% of the patients after disclosure of the 2-[^18^F]FDG-PET results [42, 43, 60]. However, the changes in patient management are dependent on the diagnostic performance of the clinicians before the biomarkers are added. In our study, the clinicians had a high accuracy in diagnosing AD patients and therefore the changes may be bigger in a study with less experienced clinicians. Nevertheless, addition of CSF biomarkers significantly reduced the need for ancillary investigations for correct diagnosed AD patients compared to addition of 2-[^18^F]FDG-PET, which underlines the role of CSF biomarkers in confirming the AD diagnosis.

There are some limitations to our study. First, evaluations and interpretations of biomarkers were deliberately based on clinical routine procedures and locally determined cut-points, which may lessen the generalization to other centers in the absence of a standardized clinical application of the biomarkers. Furthermore, we did not use the CSF Aβ42/Aβ40 ratio, which has been shown to increase the overall accuracy of the CSF analysis [62], because this analysis was not performed when this study population was included.

Also, patients in this study were included based on the clinical indication for both 2-[^18^F]FDG-PET and CSF biomarkers, which resulted in a high level of drop-outs during the retrospective selection process (S1 Fig) and clearly induces a selection bias. Moreover, the non-AD group included a heterogenous group of patients diagnosed with either DLB, FTD, VaD, other diagnosis, or SCD at 12 months follow-up. On the other hand, our study population consisted of patients with diagnostic uncertainty and need for further investigations, which is the clinically relevant subgroup to investigate the clinical value after addition of the biomarkers. Furthermore, there is a risk of circularity as the results of 2-[^18^F]FDG-PET and CSF biomarkers were included as part of the follow-up clinical evaluation.

Finally, we cannot exclude the possibility of a potential incorporation bias towards the biomarkers, which may be reflected in a greater change in patient management and confidence in diagnosis after addition of the biomarker.

The strength of the study was that the diagnostic process with addition of biomarkers to the standard diagnostic program reflected a routine clinical setting. Moreover, we included patients from a memory clinic instead of a selected research population, and the patients had a clinical indication for ancillary investigation with 2-[^18^F]FDG-PET and CSF biomarkers.

To conclude, we found that 2-[^18^F]FDG-PET had a comparable clinical impact to CSF biomarkers on diagnosis, but CSF biomarkers seemed to have a more significant clinical impact on corroborating the diagnosis of AD. This was demonstrated by a slightly higher diagnostic classification accuracy together with a higher diagnostic confidence and a reduced need for ancillary investigations for correctly classified AD as compared to addition of 2-[^18^F]FDG-PET.

The findings in this study are in line with the existing studies that indicate that both 2-[^18^F]FDG-PET and CSF biomarkers have a clinical value in diagnosing patients suspected of dementia. Furthermore, our finding of slightly higher clinical value after addition of CSF biomarkers in patients suspected of AD, adds some knowledge to the first choice of two common biomarkers in diagnostic algorithms, as most memory clinics have implemented a stepwise approach of the diagnostic work-up. However, it may be the case that our findings reflect sample-specific characteristics. Future studies should compare the clinical impact of 2-[^18^F]FDG-PET and CSF biomarkers in a larger study with less experienced clinicians, in addition to an evaluation of the cost-effectiveness and patients’ experience with the biomarkers to optimize the clinical utility of biomarkers.

Furthermore, the clinical impact of 2-[^18^F]FDG-PET may increase as several fully and semi-automatic tools have been developed to assist the visual assessment [63].

## Supporting information

S1 Fig Flow diagram of the study population. (DOCX)

S1 Table Baseline demographics for the retrospective cohort and the PredictND cohort. (DOCX)

S2 Table The clinical impact of 2-[^18^F]FDG-PET and CSF biomarkers on diagnosis and confidence in diagnosis for AD, DLB, and FTD. (DOCX)
