# Supplementary material for: Comparison of the clinical impact of 2-[18F]FDG-PET and cerebrospinal fluid biomarkers in patients suspected of Alzheimer’s disease
Source: PLoS One. 2021 Mar 12;16(3):e0248413. doi: 10.1371/journal.pone.0248413 (PMC7954298; doi:10.1371/journal.pone.0248413)
Supplement: S1 Fig — (DOCX) [file pone.0248413.s001.docx]

**S1 Figure. Flow diagram of the study population**


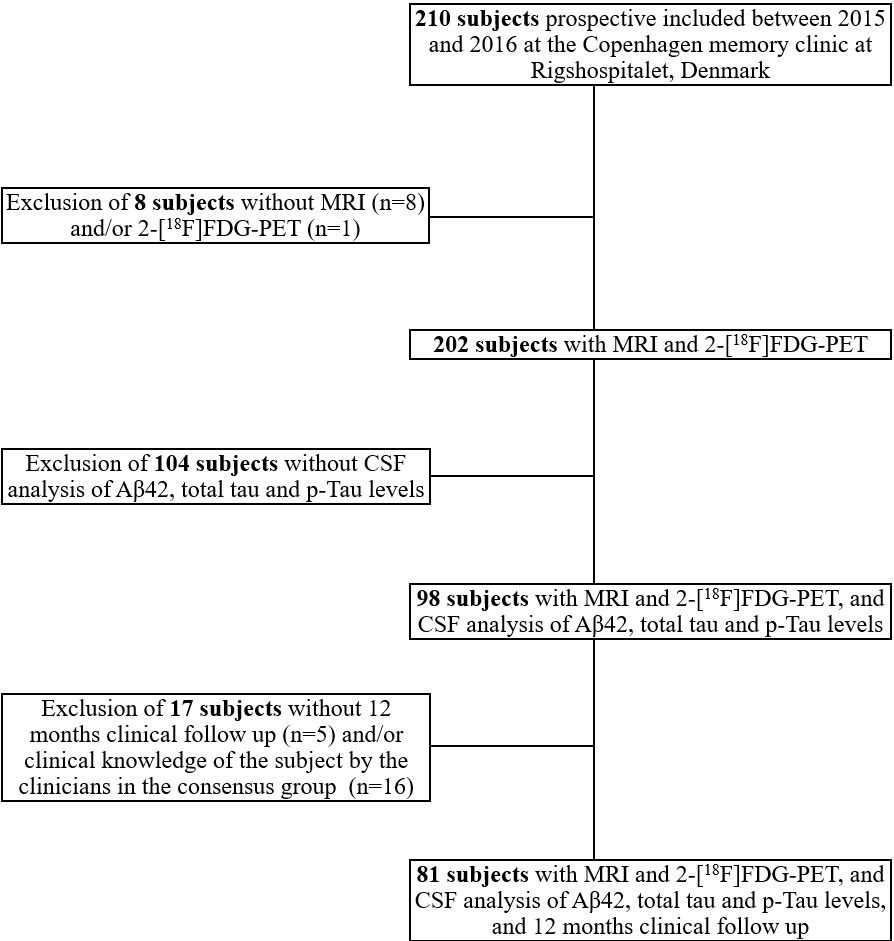
Abbreviations: Aβ42: amyloid beta 1-42; CSF: cerebrospinal fluid; MRI: magnetic resonance imaging; n: number; p-tau: phosphorylated tau at threonine 181; 2-[^18^F]FDG-PET: 2-[^18^F]Fluoro-2-deoxy-D-glucose positron emission tomography;
