# Supplementary material for: Comparison of the clinical impact of 2-[18F]FDG-PET and cerebrospinal fluid biomarkers in patients suspected of Alzheimer’s disease
Source: PLoS One. 2021 Mar 12;16(3):e0248413. doi: 10.1371/journal.pone.0248413 (PMC7954298; doi:10.1371/journal.pone.0248413)
Supplement: S1 Table — (DOCX) [file pone.0248413.s002.docx]

**S1 Table. Baseline demographics for the retrospective cohort and the PredictND cohort**

|  | Included patients from the retrospective cohort | PredictND cohort |
| --- | --- | --- |
| Patients, n | 81 | 210 |
| Female, n (%) | 40 (49) | 111 (53) ^a^ |
| Age, means years ±SD | 69 (±9) | 71 (±10) ^a^ |
| Education, mean years ±SD | 13 (±2.9) | 13 (±2.9) ^a^ |
| MMSE, median score (range) | 27 (19-30) | 28 (18-30) ^a^ |
| Syndrome diagnosis, n (%) (SCD/MCI/dementia/missing) | 15 (19)/ 13(16)/ 53 (65)/ 0 | 52 (25)/ 46 (22)/ 109 (51)/ 3(2) |
| Etiology diagnosis, n (%) (AD/non-AD/missing) | 37 (46) /44 (54)/ 0 | 90 (43)/ 118 (56)/ 2 (1) |
| Etiology diagnosis, n (%) (AD/DLB/FTD/VaD/other diagnosis/SCD/missing) | 37 (45)/ 5 (6)/ 3 (4)/ 6 (7)/ 15 (19)/ 15 (19)/ 0 | 90 (43)/ 8 (4)/ 6 (3)/ 21 (10)/ 22 (10)/ 52 (25)/ 11 (5) |

Abbreviations: AD: Alzheimer's disease; DLB: dementia with Lewy bodies; FTD: frontotemporal dementia; MCI: mild cognitive impairment; MMSE: mini-mental state examination; n: number; SCD: subjective cognitive decline; SD: standard deviation; VaD: vascular dementia

^a^Data missing for 2 patients
