# Supplementary material for: Comparison of the clinical impact of 2-[18F]FDG-PET and cerebrospinal fluid biomarkers in patients suspected of Alzheimer’s disease
Source: PLoS One. 2021 Mar 12;16(3):e0248413. doi: 10.1371/journal.pone.0248413 (PMC7954298; doi:10.1371/journal.pone.0248413)
Supplement: S2 Table — (DOCX) [file pone.0248413.s003.docx]

**S2 Table. The clinical impact of 2-[^18^F]FDG-PET and CSF biomarkers on diagnosis and confidence in diagnosis for AD, DLB, and FTD**

| According to 12 months follow-up diagnosis, n (%) | Correct diagnose | Incorrect diagnose |
| --- | --- | --- |
| Standard diagnostic program + 2-[^18^F]FDG-PET | 38 (84) | 7 (16) |
| Change in diagnosis from standard diagnostic program | 0 | 1 |
| Standard diagnostic program + CSF biomarkers | 40 (89) | 5 (11) |
| Change in diagnosis from standard diagnostic program | 3* | 3 |
| Diagnostic confidence, mean VAS score ±SD | | |
| Standard diagnostic program + 2-[^18^F]FDG-PET | 83±11 | 63±18 |
| Change in diagnostic confidence from standard diagnostic program | 9±11**^×^** | -4±16 |
| Standard diagnostic program + CSF biomarkers | 88±10 | 71±19 |
| Change in diagnostic confidence from standard diagnostic program | 14±11**^×^*** | 7±17 |

Abbreviations: AD: Alzheimer's disease; CSF: cerebrospinal fluid; DLB: dementia with Lewy bodies; FTD: frontotemporal dementia; n: number; SD: standard deviation; standard diagnostic program: medical history, physical and neurological examinations, cognitive testing, routine blood screening, and MRI; VAS: visual rating scale; 2-[^18^F]FDG-PET: 2-[^18^F]Fluoro-2-deoxy-D-glucose positron emission tomography;

**^×^**Statically significant difference as compared to standard diagnostic program (p <0.05).

*Statically significant difference as compared to 2-[^18^F]FDG-PET (p <0.05).
